# Cabbage (*Brassica oleracea* var. *capitata*) Development in Time: How Differential Parenchyma Tissue Growth Affects Leafy Head Formation

**DOI:** 10.3390/plants13050656

**Published:** 2024-02-27

**Authors:** Zihan Liu, Jorge Alemán-Báez, Richard G. F. Visser, Guusje Bonnema

**Affiliations:** Plant Breeding, Wageningen University and Research, 6708 PB Wageningen, The Netherlands; zihan.liu@wur.nl (Z.L.); jorge.alemanbaez@wur.nl (J.A.-B.); richard.visser@wur.nl (R.G.F.V.)

**Keywords:** *Brassica oleracea*, cabbage, rosette leaves, heading leaves, palisade parenchyma, spongy parenchyma, leaf curvature, abaxial/adaxial differential growth

## Abstract

This study aims to categorize the morphological changes during cabbage (*B. oleracea* ssp. *capitata*) development, seedling, rosette, folding, and heading, and to elucidate the cellular mechanisms of the leaf curvature, essential for the formation of the leafy head. We followed the growth of two cabbage cultivars with distinct head shapes (round and pointed) and one non-heading collard cultivar; we phenotyped the size and volume of the whole plant as well as the size, shape, and curvature of the leaves during growth. By integrating these phenotypic data, we determined the four vegetative stages for both cabbages. The histological phenotypes of microtome sections from five distinct leaf positions of the rosette, folding, and heading leaves at two timepoints during leaf growth were quantified and revealed variations in cellular parameters among leaf types, between leaf positions, and between the adaxial and abaxial sides. We identified two synergistic cellular mechanisms contributing to the curvature of heading leaves: differential growth across the leaf blade, with increased growth at the leaf’s center relative to the margins; and the increased expansion of the spongy parenchyma layer compared to the palisade parenchyma layer, resulting in the direction of the curvature, which is inwards. These two processes together contribute to the typical leafy heads of cabbages.

## 1. Introduction

Cabbage (*Brassica oleracea* L. var. *capitata*) is an important leafy vegetable consumed worldwide, with a global production of 71 million tons in 2021 (fao.org/faostat, accessed on 5 December 2023). The economically important part of cabbages is the leafy head, a healthy source of fiber, minerals, and vitamins [1]. The leafy head is an important agronomic trait that facilitates transport and storage. This trait has also been selected in other crops, such as Chinese cabbage (*Brassica rapa* L. ssp. *pekinensis*), radicchio (*Cichorium intybus*), and lettuce (*Lactuca sativa*). To date, little is known about the leafy head trait in cabbage. Most research on the leafy head formation in *Brassicas* has been done on Chinese cabbage [2,3,4,5]. Despite the different head shapes and leaf architecture (more pronounced large flat midribs in Chinese cabbage compared to cabbage), cabbage and Chinese cabbage have a similar overall development and go through four vegetative stages to form the leafy head: the seedling, rosette, folding, and heading stages. These stages have not been precisely defined yet in cabbage, unlike in Chinese cabbage [4,6,7,8]. In cabbage, Alemán-Báez et al. [7] provided an overall characterization of the rosette and heading stages, while Zhang et al. [8] identified a transition stage occurring between these stages in both cabbage and Chinese cabbage. In Chinese cabbage, Wang et al. [6] and Sun et al. [4] determined the four vegetative stages by characterizing the size, shape, and curvature of the leaves that the plants produce. At the seedling stage, Chinese cabbage plants produce small round leaves with flat or downward curved blades. These seedling leaves have a long and thin petiole. At the rosette stage, the plant produces large round leaves with flat blades. These leaves are organized around the stem to form a rosette. At the folding stage, the plant produces leaves with upward-curved blades and wider petioles. At the heading stage, the plant produces extremely inward-curved leaves without a petiole but a wide midvein that starts overlapping and wrapping around the shoot apical meristem (SAM) to form the leafy head [4]. The outer heading leaves constrain the inner heading leaves to fill the head. The seedling, rosette, folding, and outer heading leaves are involved in photosynthesis, whereas inner heading leaves do not photosynthesize. Instead, they serve as nutrient storage organs [4,6]. A recent study has indicated that leaf veins play a critical role as a structural framework in Chinese cabbage leafy head formation, and the distribution of auxin in specific areas of leaf veins is a key factor in leaf curvature and, thus, head formation [9].

To study leafy head development and thus to understand leaf curvature, we need to understand the leaf cellular structure, as cell sizes and shapes define organ sizes and shapes. The leaf cells are arranged as a sandwich, with two outer epidermal layers surrounding the internal mesophyll tissue [10]. The upper epidermal layer is covered with a waxy layer that protects the leaf against biotic and abiotic stresses, while the lower epidermis layer contains most of the stomata involved in gas and water exchange [10]. Meanwhile, the mesophyll tissue represents the main site of photosynthesis in the leaves. For most plants, the mesophyll tissue is divided into two tissue types: the palisade parenchyma, located beneath the upper epidermis layer and the spongy parenchyma, above the lower epidermis layer. The palisade parenchyma consists of long cylindrical cells tightly arranged in a vertical orientation that facilitates the absorbance of photons by the many chloroplasts for photosynthesis [10]. Meanwhile, the spongy parenchyma consists of cells with irregular shapes arranged in a loose way. The large intracellular space facilitates gas exchange (O_2_, CO_2_) during both respiration and photosynthesis processes and water evaporation. Moreover, within the spongy parenchyma the vessels are located, with the xylem transporting water and minerals to the leaves, and the phloem distributing the photosynthetic products to other parts of the plant [10].

In both cabbage and Chinese cabbage, seedling and rosette leaves are flat, while folding and heading leaves are curved upwards and inwards [4,7]. Mathematical and biophysical models of the *cin* mutant of *Antirrhinum majus* have provided evidence that the curvature of the leaf blades results from differential cell growth rates between the leaf central region and the margins [11]. Moreover, *CINCINNATA (CIN)* encodes a TEOSINTE BRANCHED/CYCLOIDEA/PCF (TCP) protein, an orthologue of which was identified to regulate the leafy head shape in Chinese cabbage, also through differential growth between the central and marginal leaf regions [5]. A relatively high expression of *BrpTCP4* genes in the marginal regions arrests cell division, while a low expression in the central regions enhances the cell division necessary for the overall incurvature of the head leaves. In 2016, Cheng et al. [12] studied the genomic differences between heading and non-heading morphotypes in both *B. oleracea* and *B. rapa*. They identified selective sweeps associated with the leafy head formation. Inside these regions, several candidate genes related to leaf polarity determination, including *ARF3/4*, *KANADI*s, and a member of the *HD-ZIP III* gene family, were identified. Moreover, Liang et al. [13] provided further genetic evidence that the allelic variation of these genes was associated with head traits in Chinese cabbage. They also identified that the *RDR6* gene, which is involved in trans-acting short interfering RNAs (tasiRNAs)’s biogenesis regulating *ARF3*/*4* transcript abundance, plays a role in the formation of the leafy head [14]. Ren et al. [15] identified that the *BcpLH* (*Brassica rapa* ssp. *pekinensis LEAFY HEADS*) gene, a close homologue of *HYL1* (*HYPONASTIC LEAVES 1*) in *A. thaliana*, was involved in the leafy head formation in Chinese cabbage. *HYL1* is involved in the biogenesis of micro-RNAS (miRNAs) [16], which corroborates with the many genes involved in leafy head formation that are regulated by small RNAs. These studies suggest that genetic elements involved in adaxial and abaxial leaf polarity determination and the regulation of growth (cell division and cell expansion) participate in leaf curvature and leafy head formation. 

For further genetic studies of the leaf curvature and leafy head formation in cabbage, a detailed description of its development is essential. This will help define the different developmental stages and the growth patterns of corresponding leaves. This study aims to determine the developmental stages of cabbage and identify the phenotypic differences between leaves produced at these stages, both at the whole plant/whole leaf level and the cellular level. To determine the cabbage vegetative stages, we followed the vegetative growth of two heading cabbages with contrasting head shapes (round and pointed) and compared it with the vegetative growth of a non-heading collard green (*B. oleracea* var. *acephala*). From both cabbages, we scored the leaf size, shape, and curvature at different time points within the vegetative stages. Moreover, we selected a set of leaves to follow their development *in planta*. Using microtome dissection, we identified variations in cellular structure between different leaf positions and between adaxial and abaxial sides. We hypothesize that the differential growth between the central and marginal regions, as well as between the adaxial and abaxial sides of the leaves, causes the typical leaf curvature seen in the leafy head.

## 2. Materials and Methods

### 2.1. Plant Materials and Growing Conditions

Three *B. oleracea* hybrid cultivars were utilized in this study: a round heading cabbage (cv Excalibur, Bejo Zaden, Warmenhuizen, The Netherlands), a pointed heading cabbage (cv Sonsma, Rijk Zwaan Zaadteelt en Zaadhandel B.V., De Lier, The Netherlands), and a non-heading collard green (cv Teddy, Chiltern Seeds, Wallingford, UK). The two cabbage cultivars were selected for their contrasting leafy head shape, and the collard cultivar for its close phylogenetic relationship with the heading cabbages [17]. Seeds from these cultivars were sown in germination trays with sandy soil in the Unifarm greenhouse at Wageningen University and Research (51°59′11″ N latitude, 05°39′52″ E longitude) at two timepoints: in the last week of March 2020 and in the last week of August 2020. The seedlings used in both experiments were cultivated in the same greenhouse compartment with a 16 h day and 8 h night cycle. However, there were clear variations in weather conditions between the two experiments. In March, the average temperature was 18.5 °C and the relative humidity was 70%, while in August, the average temperature was 23.1 °C and the relative humidity was 60%. Two weeks after sowing (for both experiments), the seedlings were transplanted into 2 L pots with potting compost (Lentse potgrond No. 2) and placed within the same greenhouse following a Randomized Complete Block Design (RCBD) with three blocks. Each of these blocks served as a biological replicate.

### 2.2. Cabbage Plants and Leaf Development in Planta and Histological Studies

This experiment started in March 2020 and included, in total, eighteen plants, nine for round cabbage and nine for pointed cabbage, divided over 3 blocks (Table 1). Each block contained three round and three pointed cabbage plants. At 30 DAS, the two most similar plants per plot from both the round and pointed cabbage were selected to conduct two non-destructive experiments: one plant was utilized to follow both leaf and whole plant development from seedling to leafy head formation *in planta*, while the other plant was utilized to perform histological studies of leaf tissue samples (Table 1).

**Figure 1:**
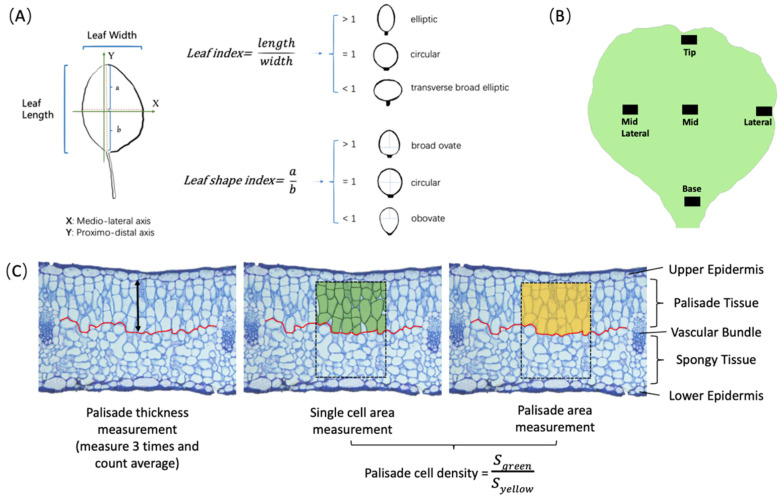
Description of leaf development traits generated *in planta* and histological traits. (**A**) The definition of leaf index and leaf shape index jointly define the leaf shape. (**B**) Illustration of the sampling position for the histological study. (**C**) Data extraction strategy for tissue thickness and cell density. The red line is drawn manually to separate palisade tissue and sponge tissue according to the shape of the cells. The palisade tissue thickness is the distance from the upper epidermis to the red line. The spongy tissue thickness is the distance from the lower epidermis to the red line. The rectangular dashed frame is the area of 125,600 μm^2^. The formula of cell density for palisade tissue is depicted in the figure and is similar for cell density of spongy tissue.

For the whole plant development experiment, the aerial part of the round and pointed cabbages was photographed from top and side views at weekly intervals from 30 DAS to 126 DAS. For the leaf shape development *in planta* experiment, we phenotyped selected leaves by drawing their circumference on a sheet of paper to calculate the leaf blade area, leaf index, and leaf shape index (Figure 1A). The terms used to characterize the shape of the cabbage leaf are derived from the Community Plant Variety Office [18] (Figure 1A). To ensure uniformity in leaf size comparisons between the round and pointed cabbages, we selected the 4th (seedling) and 15th (rosette) leaf in the round cabbage and the 5th (seedling) and 15th (folding) leaf in the pointed cabbage at different time points (Table 1). For the leaf histological experiment, we collected leaf tissue from around the 15th and 21st leaves at five defined positions along the leaf blade (Figure 1B) at two time points (t1 and t2) (Table 1). The t2 sample was collected from a position that is axially symmetrical to the location of the t1 sample. Each tissue sample (4–8 mm^2^) was carefully excised using a scalpel to include the complete transverse section, including both epidermal layers and the mesophyll tissue. The excised tissue samples were immediately fixed in 5% glutaraldehyde in 0.1 M phosphate buffer, at pH 7.2, and left overnight at 4 °C. The samples were washed four times for 15 min in 0.1mM phosphate buffer and two times for 15 min in Milli-Q water. The samples were then dehydrated through a series of increasing ethanol concentrations (10, 30, 50, 70, 96, and 100%) for 20 min at each step. Following dehydration, the samples were infiltrated and embedded using the Technovit® Glycol Methacrylate 7100 Kit (Kulzer, Hanau, Germany). Embedded samples were sliced into 7 μm transverse sections and stained with toluidine blue. These stained sections were then photographed using a camera mounted on a microscope (ZEISS AxioCam ICc5, Jena, Germany). These photographs were utilized to measure the cell number, cell size, and cell density of both palisade and spongy parenchyma (Figure 1C) using ImageJ (Version: 2.0.0-rc-43/1.50e) software [19]. The cell number is determined by counting the cells within a specified area of 125,600 μm^2^ (to include approximately 50 cells), as indicated by the rectangular dashed frame in Figure 1C. In this frame, cells are included in the count if more than half of their area is within the frame. The cell area is calculated as the average area of all complete cells within this area. Cell density refers to the proportion of tissue occupied by cells, with the intercellular space accounting for the remaining portion of the tissue within the frame (Figure 1C). In addition, we measured the thickness of the palisade and spongy mesophyll layers and calculated the ratio of the palisade-to-spongy parenchyma (P/S) for the tissue thickness, cell area, and cell density at both t1 and t2. We also calculated the ratio of the change in the palisade to the change in the spongy parenchyma parameters between t1 and t2 (∆P/∆S = (P_t2_ − P_t1_)/(S_t2_ − S_t1_)).

### 2.3. Leaf Morphology Studies

**Figure 2:**
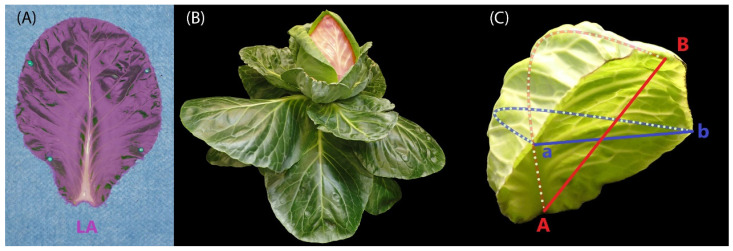
Cabbage traits scored in the cabbage leaf morphology studies. (**A**) Visualization of the leaf area on a rosette leaf in a pointed cabbage. (**B**) Visualization of the first heading leaf (in red) in a pointed cabbage. (**C**) Visualization of the methodology utilized to calculate the leaf curvature ratio. The straight red line indicates the closest distance between the proximal and distal edges, while the blue line is between the lateral edges. The dotted red line follows the proximal–distal curvature, and the blue line the lateral curvature. A and B are the base and tip points of the leaf’s proximal–distal axis; a and b are the left and right points of the leaf’s medio-lateral axis.

This experiment started in August 2020 and included in total 90 plants, 30 for each cultivar: round cabbage, pointed cabbage, and collard green. From each of these cultivars, 10 plants were placed in each of the three blocks. At weekly intervals from 28 to 77 DAS (Table 1), one plant per block of each cultivar was randomly selected to score the complete number of leaves and calculate the area of each single leaf. The total number of leaves included the number of scars on the stem produced by leaves that senesced. To calculate the leaf area, the complete set of leaves was detached from the stem, positioned from oldest to youngest, and photographed. The youngest leaf we phenotyped was 4 cm in length. The leaf area (Figure 2A) was extracted from these photographs using ImageJ (Version: 2.0.0-rc-43/1.50e) software. When the cabbage plants (round and pointed) showed a leafy head, the position (from oldest to youngest) of the first heading leaf was scored. The first heading leaf is defined as the oldest (outermost) leaf that was part of the leafy head (Figure 2B). Additionally, the degree of leaf curvature in two axes (proximo-distal and medio-lateral) was assessed for the complete set of leaves of both round and pointed cabbages at 70 DAS, a stage when both cabbage cultivars showed a leafy head. For this, a leaf curvature ratio was calculated for each axis, dividing the shortest distance between the two opposing leaf edges (straight line) by the distance following the curvature of the leaf (dotted line) (Figure 2C). 

## 3. Results

### 3.1. Whole Plant Development

**Figure 3:**
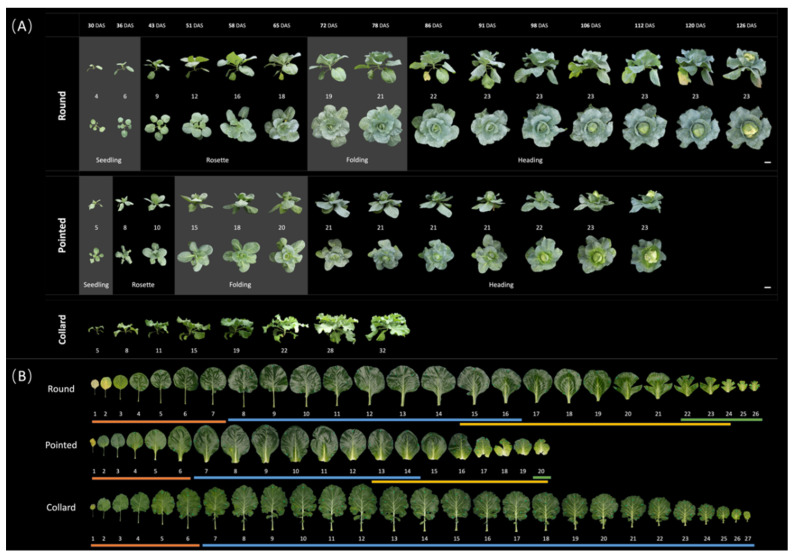
Plants and leaves of both round and pointed cabbage and collard green at four developmental stages. (**A**) Overview of round and pointed cabbage development process from the top and side view. Collard green was recorded as control only from the side view. Photos were taken once a week. Leaf numbers of the plants at each time point were put under the side-view photos. The separation of different developmental stages is indicated by the grey blocks: seedling, rosette, folding and heading stages. DAS = Days After Sowing. Scale bars represent 11 cm. (**B**) All leaves from round cabbage, pointed cabbage, and collard green plants were harvested 77 DAS. The leaf numbers are under the photos. The lines under the leaf numbers represent leaves of different developmental stages: orange (seedling stage), blue (rosette stage), yellow (folding stage), and green (heading stage).

At weekly intervals from 30 to 126 days after sowing (DAS), plants from the three *B. oleracea* cultivars (round cabbage, pointed cabbage, and collard green) were photographed from both top and side views. In addition, leaves were removed and photographed (see Section 2.2 and Section 2.3). This provided information about whole plant growth, including the leaf number (Table S1), as well as the leaf shape and size (Table S2). Based on both the whole plant phenotype (Figure 3A) and their detached leaves (Figure 3B), we determined four vegetative stages for both round and pointed cabbages: seedling, rosette, folding, and heading.

At the seedling stage, when both cabbage plants were small (Figure 3A), the internodes between the seedling leaves were relatively large. Both round and pointed cabbage seedling leaves were small and round. These leaves were flat or downward curved with a petiole. However, in the pointed cabbage, the petioles of the younger seedling leaves were reduced compared to the seedling leaves of the round cabbage (Figure 3B). The seedling stage in the round cabbage (until 36 DAS) lasted longer than in the pointed cabbage (until 30 DAS) (Figure 3A,B).

At the rosette stage, the cabbage plants’ growth was evident, generating new leaves and expanding their leaves. The internodes between leaves decreased to establish a rosette structure. In the round cabbage, the rosette stage was from ~43 to 65 DAS, and the rosette leaves had a “broad ovate” shape with a reduced petiole (Figure 3A,B). In pointed cabbage, the rosette stage was earlier and shorter, from ~36 to 43 DAS, and the rosette leaves showed an “obovate” shape without a petiole (Figure 3A,B).

During the folding stage, the increase in the volume of both the round and pointed cabbage plants decelerated. The plants from the cabbages (round and pointed) produced upward curved leaves without petioles (Figure 3A,B). In round cabbage plants, the folding stage was from ~72 to 78 DAS, while in pointed cabbage plants, it was from ~51 to 65 DAS (Figure 3A,B).

At the heading stage, the cabbage plants produced leaves with an extreme inward curvature that caused their overlapping around the shoot apical meristem (Figure 3A). The outer heading leaves constrained the inner heading leaves, forcing them to fill the leafy head; however, these leaves also still expanded. The heading stage in round cabbages started at 70 DAS, while in the pointed cabbage, it started at 56 DAS (Figure 3A,B). At the heading stage, the seedling leaves had senesced, while the rosette leaves all stayed green.

For collard plants, only two distinct developmental stages were identified: the seedling stage and the rosette stage (Figure 3A). Once the rosette stage was reached, the morphology of both the plants and their leaves remained consistent. The leaves were elliptic, exhibited characteristic wavy blades, and possessed elongated petioles.

### 3.2. Changes in Leaf Morphology during Plant Development

To identify changes in leaf morphology during the four vegetative developmental stages (seedling, rosette, folding, and heading), we followed the development of cabbage plants from 28 to 77 DAS. At weekly intervals, we scored the total leaf number and conducted analyses of their size, curvature, and shape (see Section 2.3). This provided information concerning the time intervals between the formation of each leaf, their growth speed, and their final shape and curvature (Tables S1–S3). Based on these combined data, we described the seedling, rosette, folding, and heading leaves for both cabbages. 

**Figure 4:**
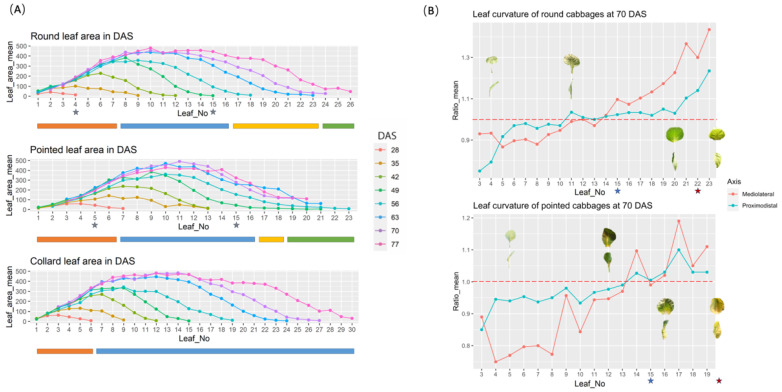
Description of leaf area and leaf curvature. (**A**) Leaf area from round cabbage, pointed cabbage, and collard green plants at different DAS. The stars indicate the number of the sampled leaves for leaf shape analysis. The bars under each figure represent developmental stages: orange (seedling stage), blue (rosette stage), yellow (folding stage), and green (heading stage). (**B**) Assessment of the curvature of leaves from round and pointed cabbage plants at 70 DAS. The red dashed line represents the ratio mean equal to 1.0, which means the leaf is flat. The stars indicate the number of sampled leaves for the histological study.

Seedling leaves in round cabbages include the first seven leaves, and in pointed cabbages, the first six leaves (Figure 3B and Figure 4A,B). In both cabbages, each successive seedling leaf reached a larger final size, and their growth ceased after 49 DAS (Figure 4A). Moreover, in both round and pointed cabbages, the older seedling leaves curved downward (curvature ratio < 1), while the younger ones were flatter (curvature ratio ≈ 1) (Figure 4B). 

Rosette leaves in round cabbage plants included leaves 8 to 16, and in pointed cabbage leaves, 7 to 16 (Figure 3B and Figure 4A,B). These rosette leaves represent the largest leaves of both cabbage plants and cease growth at 70 DAS for round cabbage plants and 63 DAS for pointed cabbages (Figure 3B and Figure 4A). In both cabbage cultivars, the older rosette leaves curve slightly downward, the middle rosette leaves are flat, while the youngest rosette leaves curve slightly upward (Figure 4B). 

The folding leaves in the plants of both cabbage cultivars start at leaf 17 until the first heading leaf (Figure 3B and Figure 4A,B). Although the folding leaves clearly show an upward curvature (curvature ratio > 1) (Figure 4B), they do not yet overlap to form a mold for the leafy head. 

The first heading leaf for round cabbages was leaf 24, while it was leaf 18 for the pointed cabbage. These heading leaves show inward curvature, which is stronger in the round cabbage plants compared to the pointed cabbage plants (Figure 4B).

**Figure 5:**
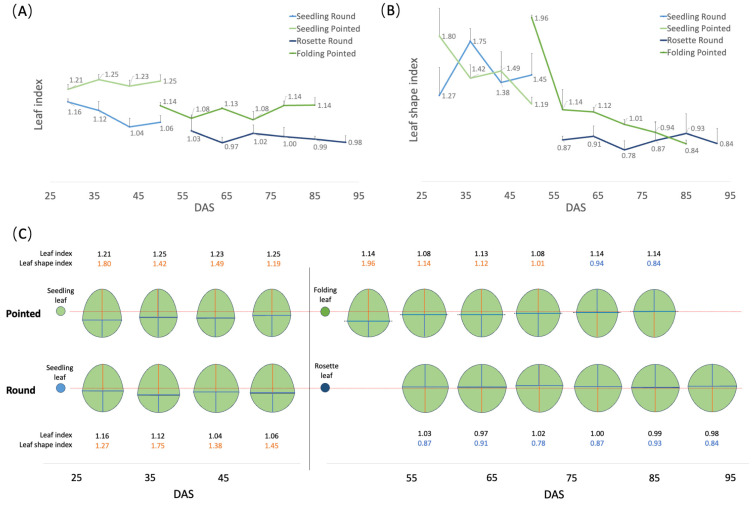
Description of leaf shape of round and pointed cabbages. (**A**) Changes in leaf index of the seedling, rosette, and folding leaves over time. (**B**) Changes in leaf shape index of the seedling, rosette, and folding leaves over time. (**C**) Leaf shape simulation diagram based on the values from (**A**,**B**). The red lines indicate the position of the medio-lateral axis at the middle of the proximo-distal axis (leaf shape index = 1.0). The orange number means the leaf shape index is larger than 1.0, while the blue number means it is smaller than 1.0.

To investigate the dynamic changes in the leaf shape during leaf growth more accurately, two leaves for each of three cabbage plants of both cultivars (round and pointed) were selected to phenotype at weekly intervals while they remained attached to the plant (see Section 2.2). For the round cabbage, we selected a seedling and a rosette leaf, while for the pointed cabbage, a seedling and a folding leaf were selected. For these leaves, we calculated both the leaf index and the leaf shape index (Table S4) (see Section 2.2, Figure 1A) as indicators of their shape (Figure 5A,B).

Both round and pointed seedling leaves have an elliptical shape (leaf length/width > 1), with the widest part at the proximal base (broad-ovate shape) (Figure 5C). In round cabbage seedling leaves, the proximal base was wider than in pointed cabbages. The rosette leaves of round cabbages are circular (leaf length/width = 1), with the widest part at the distal end (obovate shape) (Figure 5C). The folding leaves of pointed cabbages are elliptical like their seedling leaves (leaf length/width > 1), and the widest part of the leaf transitions from the proximal base (broad ovate shape) in younger leaves to the distal end (obovate shape) in older leaves (Figure 5C).

### 3.3. Defining the Developmental Stages of Cabbage

To define the start and the duration of each developmental stage (seedling, rosette, folding, and heading) in both round and pointed cabbages, we integrated the data of whole plant development (Figure 3), leaf size (Figure 4A), and leaf curvature (Figure 4B). For both round and pointed cabbages, the heading stage, which extends to head maturity, is the longest, while the folding stage is the shortest (Table 2). For the early-maturing pointed cabbage variety, each stage is shorter than that of round cabbage, and this is especially the case for the folding stage, with fewer folding leaves. The number of the first heading leaf and, thus, the onset of the heading stage differs between plants from the same variety in the same greenhouse (Table S5), showing that leafy head formation is affected by small environmental fluctuations. 

### 3.4. Histology of Leaf Development

#### 3.4.1. Difference among Leaf Positions

To investigate the morphological changes occurring at the cellular level during leaf development in both round and pointed cabbage leaves, we phenotyped the rosette leaf of the round cabbage, the folding leaf of the pointed cabbage (previously used for assessing the leaf shape, see Section 3.2), and heading leaves from both cabbages. From these leaves, we sampled the tissue from five positions (tip, mid, base, mid-lateral, and lateral) at two time points: t1 and t2, with a four-week interval between them (see Section 2.2) to assess the cell number, cell area, cell density, and tissue thickness (Table S6).

**Figure 6:**
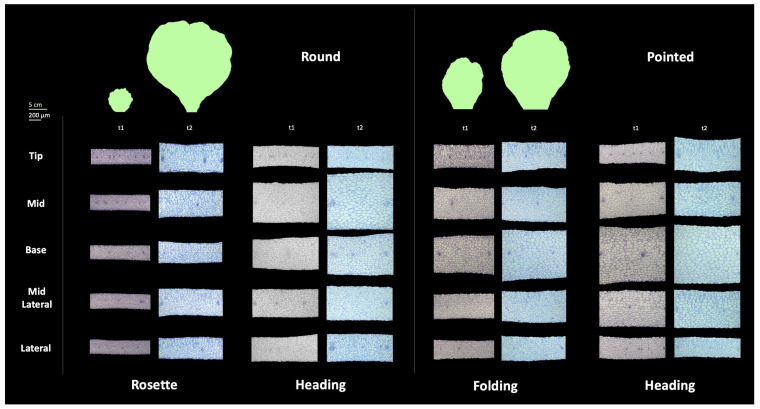
Leaf transverse sections of round and pointed cabbages. Leaf tissue from the rosette and heading leaves of round cabbage and the folding and heading leaves of pointed were collected from five positions: tip, mid, base, mid-lateral, and lateral. Leaf tissue was collected at two time points (t1 and t2), separated by four weeks. The green leaves represent the size of the leaf at the corresponding time point. The scale bar for leaf size represents 5 cm, and for leaf transverse sections represents 200 μm.

Overall, the leaf thickness increased from t1 to t2 for all leaf positions in both round and pointed cabbages (Figure 6). This increase was largest in the rosette leaves of the round cabbage; these leaves also showed the largest increase in size. The increase in thickness was only marginal between t1 and t2 in all other leaves (round heading, folding, and pointed heading), and these leaves also showed less growth.

We also identified differences in the thickness between positions within a leaf. At t1, the rosette leaves had a comparable thickness across the positions in the round cabbage, while in both the folding and heading leaves, the thickness varied across their leaf blades. At t2, the thickness across the leaf blade in the flat rosette leaves of the round cabbage remained comparable, while in all other stages (folding and heading), the thickness varied strongly across the leaf blade. In the pointed cabbage, the base position in both the folding and heading leaves was thickest, which was already the case at t1. Interestingly, in round cabbage, the mid position of heading leaves was thicker in both t1 and t2. The position of the thickest tissue coincided with the curvature of the leaves, which was in the middle for round cabbage leaves, where bending is most extreme, and at the base of pointed cabbage folding and heading leaves, again where leaf bending is most obvious.

**Figure 7:**
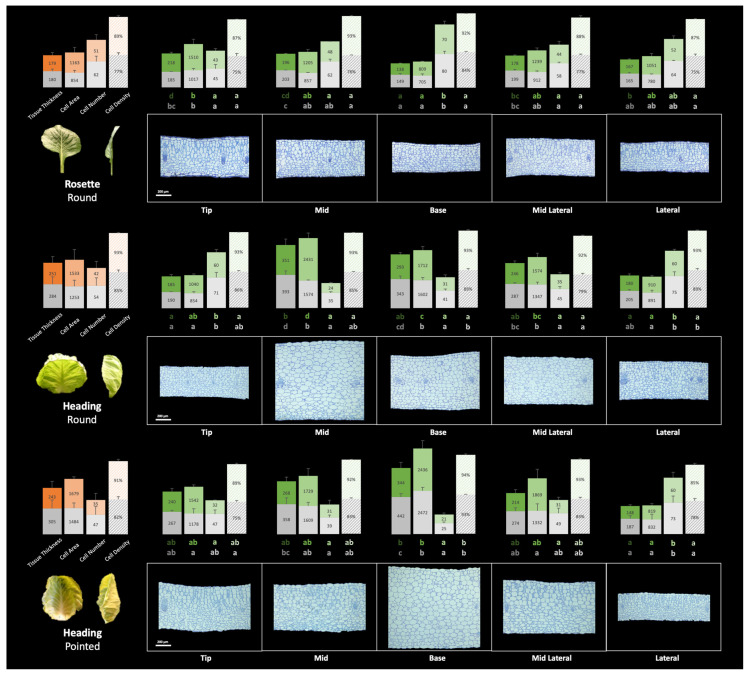
Stacked bar charts of leaf histological traits for round cabbage rosette and heading leaves and pointed cabbage heading leaf at t2. In each group of the four bars, the bars represent tissue thickness, average single cell area, cell number per 125,600 μm^2^, and cell density from left to right, respectively. The orange bar chart on the left represents the mean of the five leaf positions. The green bar charts represent the data corresponding to the five leaf positions. The top layer of the bars represents the data of palisade tissue, while the lower layer represents the data of spongy tissue. The letters under the green bars represent the groups of Least Significance Difference (LSD) with one-way ANOVA comparing specific traits across positions, with the upper letter for the data of palisade tissue, and the lower letter the data of spongy tissue. The scale bars represent 200 μm.

Although a variation in the thickness across the leaf blade was less evident in rosette leaves, the tip, mid, and mid-lateral positions were the thickest, while the base and lateral positions were the thinnest (Figure 7). The thicker mid position of the heading leaves of round cabbage was characterized by a relatively low number of cells of a large size (large cell area). The cell area and cell number were inversely correlated, so when cells were large, their numbers were low and vice versa. In contrast to these very large mid-position cells, the cells at the base and mid-lateral position were intermediate, while at the tip and lateral positions, the cells were smaller. 

Similar to the heading leaves in round cabbages, the thickest base position of the heading leaves of the pointed cabbage was characterized by the largest cells, while the thinner part of the leaf (the lateral position) had the highest number of cells with the smallest area. Again, the cell size and numbers were inversely correlated.

#### 3.4.2. Difference between Adaxial and Abaxial Sides

As we hypothesized that leaf curvature can be caused by both differential growth along the leaf blade and differential growth between palisade and spongy parenchyma cells (ad- and abaxial sides), we set out to compare the cellular parameters between the palisade and spongy parenchyma. The relationship of the cellular parameters between the palisade and spongy parenchyma varied between the rosette and heading leaves (Figure 7, mean values across the leaf blade). In the heading leaves of both the round and pointed cabbages, the spongy parenchyma comprised a larger proportion of the mesophyll in terms of tissue thickness and cell area compared to the rosette leaves (Figure 7).

To investigate the relationship between the difference in the palisade (P) and spongy (S) parenchyma tissue and leaf curving, we calculated the palisade-to-spongy parenchyma (P/S) ratio for the cellular parameters Tissue Thickness, Single Cell Area, and Cell Density at both timepoints (t1 and t2) to monitor changes during growth. We also calculated the ratio of the change between the palisade and spongy parenchyma from t1 to t2 (∆P/∆S = (P_t2_ − P_t1_)/(S_t2_ − S_t1_)) to evaluate the growth differences between the palisade and spongy parenchyma tissues (adaxial and abaxial sides) (Table 3). Table 3 shows the P/S and ∆P/∆S ratios averaged across the five leaf positions, while the specific ratio values (P/S and ∆P/∆S) for each position (tip, mid, base, mid-lateral, and lateral) are provided in Table S8.

The average P/S and ∆P/∆S ratios calculated across the five leaf positions in the rosette leaves of the round cabbage were around 1.0, indicating a flat leaf shape. Conversely, these ratios for the heading leaves of both the round and pointed cabbages were below 1.0, reminiscent of a curved leaf shape.

##### Tissue Thickness

The leaves grow along the three axes, the proximo-distal, medio-lateral, and adaxial–abaxial axes. Measuring the leaf thickness in transverse sections reflects the growth of mesophyll and can be quantified to define growth differences between the palisade and sponge tissue. In rosette leaves, the P/S of the tissue thickness is approximately 1.0 both at t1 and t2, while the ∆P/∆S is also close to 1.0, indicating that the growth rates of the palisade and spongy parenchyma are similar. This similarity in the growth rates of the palisade and spongy tissue contributes to the maintenance of the flat shape of rosette leaves during leaf development. However, in heading leaves of both the round and pointed cabbages, the P/S and ∆P/∆S of tissue thickness are all less than 1.0. This result suggests that the growth rate of the palisade is slower than that of the spongy parenchyma, leading to the development of inwardly curved leaves.

##### Average Single Cell Area and Cell Density

The increase in the tissue thickness results from the expansion of mesophyll cells and the enlargement of intercellular spaces, characterized by an increased cell area and decreased cell density. 

In rosette leaves, both the P/S and ∆P/∆S ratios of the single cell area were greater than 1.0, indicating a faster cell expansion rate in the palisade parenchyma compared to the spongy parenchyma (Table 3). Moreover, the P/S ratio of the cell density at both t1 and t2 is also greater than 1.0, illustrating the lower cell density in the more loosely organized spongy parenchyma tissue. Interestingly, the ∆P/∆S of the cell density is less than 0, suggesting a faster increase in the cell density in the palisade parenchyma and/or a faster decrease in the spongy parenchyma from t1 to t2. These trends are visually represented in Figure 7, where palisade parenchyma cells are larger with less intercellular space compared to spongy parenchyma. These results indicate that an increase in the intercellular space compensates for the lower cell expansion in the spongy parenchyma relative to the palisade parenchyma, resulting in comparable growth between the two tissue types. 

In the heading leaves of both round and pointed cabbages, the P/S ratio at t2 and ∆P/∆S of the cell area were all larger than 1.0, but they were less than that of rosette leaves (1.2 and 1.3 for heading leaves of round and pointed cabbages compared to 1.5 for rosette round leaves). This means that the spongy parenchyma cells expanded more in heading leaves compared to rosette leaves. The P/S ratio of the cell density at both t1 and t2 in heading leaves was similar to that of rosette leaves. In conclusion, a higher cell expansion rate and rate of increase in the intercellular space of the spongy parenchyma compared to the palisade parenchyma results in the increased growth of the spongy tissue compared to the palisade tissue in heading leaves, causing inward leaf curvature.

#### 3.4.3. Shoot Apical Meristem

**Figure 8:**
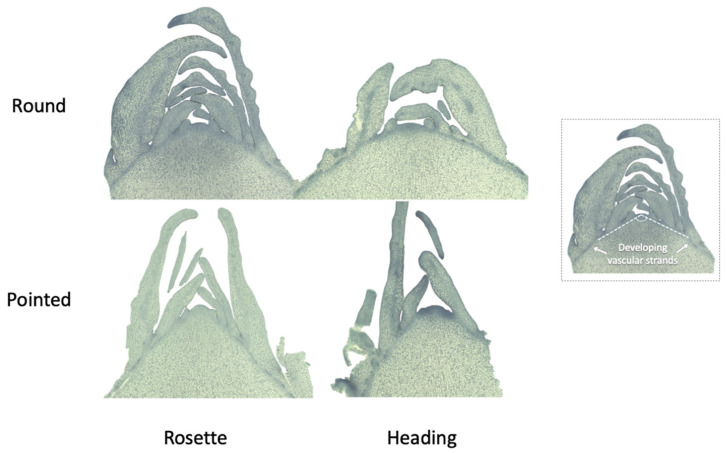
Shoot apical meristem (SAM) transverse sections of round and pointed cabbages at rosette and heading stages. At the rosette stage, SAMs were harvested at 57 DAS (round cabbage) and 43 DAS (pointed cabbage). At the heading stage, SAMs were harvested at 127 DAS (round cabbage) and 99 DAS (pointed cabbage). The angle of the developing vascular strands of the SAM is illustrated on the right side of the figure.

As the leaves developed from the shoot apical meristem (SAM), we assessed the SAM morphology. The SAM differed between the rosette and heading stages of the round cabbage, but also between the round and pointed cabbages. In round cabbages, the SAM exhibits a dome-shaped morphology, transitioning to be more flattened in the heading stage. Conversely, in pointed cabbages, the SAM is a triangular shape at the rosette stage and becomes slightly rounded in the heading stage. A notable distinction between the SAM structures of round and pointed cabbage plants is the smaller angle of the developing vascular strands observed in the pointed cabbage (Figure 8).

## 4. Discussion

Cabbages display a wide array of agronomic traits which are reflected in different cabbage types (round, red, savoy, and pointed) in the range of ecotypes (summer, autumn, and winter cabbages), in geographical locations where cabbages can be grown, and in the different market segments (fresh market, industry, and storage). The different varieties require different time periods to initiate the head formation (heading stage) and to develop tight, compact, leafy heads (head maturity). As they progress towards the heading stage, notable changes occur in the overall shape of both the cabbage plant and the size and shape of the developing leaves. This study is dedicated to studying these morphological transitions, with a special focus on the timing of each developmental stage. In addition, this study tests the hypothesis that leaf curvature, an essential step in leafy head formation, is the result of differential growth across the leaf blade and between the mesophyll cell layers. We have generated detailed phenotypes at the whole-plant level, monitoring the plant shape and volume, and at the leaf level, assessing the leaf size, shape, curvature, and cellular structure over time. By integrating the observations of both whole-plant and leaf development, we defined the seedling, rosette, folding, and heading stages in a round and pointed cabbage. The criteria used to define these developmental stages for these two types of cabbage are likely to be universally applicable to other varieties as well. 

### 4.1. Characterization and Timing of Developmental Stages

The final size and curvature of the leaves produced by both cabbages (round and pointed) change across the different developmental stages (seedling, rosette, folding, and heading). In both cabbages, the rosette leaves are the largest with a mostly flat leaf blade, while the folding leaves curve upward. Our histological study revealed that in both cabbages, the growth of rosette leaves results from both cell division and cell expansion, whereas the growth of the folding leaves, which remain smaller than rosette leaves, is mainly by cell expansion. This suggests that both cell division and expansion in cabbage leaves are co-regulated by the developmental stage. This finding is consistent with previous research, such as the study by Alemán-Báez et al. [20], which reported that miR396b and its interacting *GRF* gene, both involved in cell division during leaf development, were differentially expressed between the young rosette and heading leaves in cabbage. Similarly, Mao et al. [5] showed that the miR319–*TCP4* interaction, also regulating cell division and expansion across the leaf blade, influences both rosette leaf growth and the head shape of Chinese cabbage [5].

As cabbage plants develop from the rosette to the heading stage, there is a noticeable decrease in the leaf size coupled with an increase in leaf curvature. Folding leaves represent a transition stage, showing size and curvature that are intermediate between those of rosette and heading leaves. Interestingly, Zhang et al. [8] identified a transition stage between the rosette and heading stages by tracking gene expression changes during the development of both cabbage and Chinese cabbage. This transition stage was characterized by massive gene expression changes, with the clear upregulation of genes involved in phytohormone signaling pathways, particularly the ethylene pathway for Chinese cabbage, as well as genes predicted to regulate growth. Moreover, this transition stage was sensitive to ambient temperatures, with higher temperatures delaying the heading stage. Notably, this temperature-induced delay did not affect the expression levels of *SQUAMOSA-promoter binding protein-like* (*SPL*) genes, crucial for plant growth and development, indicating that the pathways regulating age and leafy head formation are distinct.

Moreover, we analyzed the variations between round and pointed cabbage varieties and found that the shape of the rosette leaves was indicative of the head shape; circular rosette leaves (leaf length/leaf width ≈ 1) correlated with round heads, while elliptical rosette leaves (leaf length/leaf width > 1) were associated with pointed heads. Complementary to our findings, Mao et al. [5] found in Chinese cabbage that the curvature and shape of the rosette leaves were indicative of the final head shape: flat rosette leaves corresponded with round heads, inward-curving leaves with cone-like heads, and wavy leaves with cylindrical heads. Moreover, Alemán-Báez et al. [7], across 308 cabbage accessions, and Sun et al. [21], in 152 Chinese cabbage accessions, identified significant correlations between rosette leaf and head traits. Both studies also discovered QTLs associated with both traits.

### 4.2. Differential Growth across the Leaf Blade and between the Mesophyll Cell Layers Is Associated with Leaf Curvature

**Figure 9:**
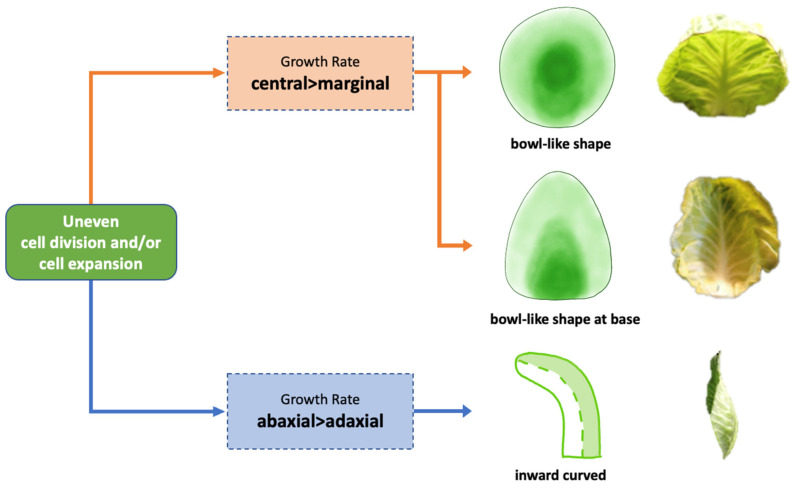
Cellular pathways regulate the formation of inward-curving leaves of cabbage heads.

Figure 9 visualizes our hypothesis that differential cell division and expansion across both the leaf blade and between leaf ad/abaxial sides results in leaf curving, which is indispensable for leafy head formation. To validate this hypothesis, we examined the cellular structure of leaf blade tissue across the leaf blade at five distinct positions (tip, mid, base, mid-lateral, and lateral) to identify variations in the tissue thickness and cell size across these areas. In the heading leaves of both cabbages, the position of the thickest leaf tissues with the largest cell sizes corresponded with the curvature’s location. In round cabbages, the mid position of the heading leaf was the thickest, while the tip and lateral were thinnest, allowing the leaf to curve at this point into a bowl shape. Conversely, in pointed cabbages, the base was the thickest and the laterals were the thinnest, which facilitated bending along the sides. 

To further test our hypothesis, we compared changes in both the palisade and spongy parenchyma layers during leaf growth in both round and pointed cabbage cultivars. 

We determined the ratio of the change in the palisade parenchyma to the change in the spongy parenchyma by analyzing the tissue thickness and cell size at two distinct time points. Subsequently, we compared these ratios between the rosette and heading leaves. The ratios observed in the heading leaves were consistently smaller than those in the rosette leaves. This observation suggests that, within the entire mesophyll, spongy parenchyma cells exhibited greater expansion, and the spongy tissue thickness increased more significantly in heading leaves that curve compared to the flat rosette leaves. This aligns with research conducted on *Arabidopsis*, demonstrating that the upwardly curved leaf morphology observed in the *hyl1* mutant is associated with abaxial epidermal cell overgrowth through cell expansion [22]. This is in agreement with a study of Yu et al. [22] in *Arabidopsis*, in which the expression level of the adaxial identity gene *REV* increased as the miR165 expression was reduced due to the absence of *HYL1* in the *hyl1* mutant. 

In our study, we did not quantify epidermal cellular growth, but the differences in growth between the palisade and spongy layers suggests that epidermal growth also differs between ad- and abaxial sides. In Chinese cabbage, the epidermal cells of heading leaf veins show an asymmetrical growth pattern, with cells on the abaxial side dividing much more rapidly than those on the adaxial side [9]. The disruption of auxin transport also induced the asymmetrical growth of the abaxial and adaxial epidermal cells in leaf veins, leading to the upward curvature of seedling leaves [9]. Studies on cabbage and Chinese cabbage have shown that the genes responsible for establishing abaxial/adaxial leaf polarity [12,13,23,24], as well as those involved in cell division and expansion [5,7] during leaf development, are crucial for the formation of the leafy head. Additionally, microRNAs that regulate these leaf development genes also play a significant role in this process [5,20,23].

## 5. Conclusions

In conclusion, our findings indicate that two synergistic cellular pathways regulate the formation of inward-curving leaves during cabbage leafy head formation (Figure 9). We clearly demonstrated that increased growth at the mid or base positions, in contrast to the lateral positions, leads to the formation of a bowl-like shape of heading leaves in round cabbages and in heading leaves bent at the leaf base for pointed cabbages. The second pathway concerns the different rates of parenchyma (palisade/spongy) cell division and expansion, resulting in the increased growth of the leaf’s abaxial side compared to the adaxial, leading to inward curvature.

## Figures and Tables

**Table 1:** Description of the experiments conducted. DAS = Days after sowing.

Sowing Date	Plants in Total	Plants Utilized in the Experiment	Type of Experiment	Experiment	Cultivars	Material Scored	Scoring Timepoints (DAS)	Traits Scored
March	18 (2 cabbage cultivars × 3 blocks × 3 plants)	3 plants per cultivar (1 per block)	Non-destructive	Whole plant development	Round and pointed cabbage	Aerial part of cabbage plants	30, 36, 43 51, 58, 65, 72, 78, 86, 91, 98, 106, 112, 120, and 126	Plant growth
				Leaf development *in planta*		4th leaf for round cabbage, 5th leaf for pointed cabbage	29, 36, 43, and 50	Leaf blade area, leaf index, and leaf shape index
						15th leaf for round and pointed cabbage	50, 57, 64, 71, 78, 85, and 92	
		3 plants per cultivar (1 per block)		Histology of leaves	Round cabbage	Leaves 15th, 15th, 15th	t1 = 57 and t2 = 85	Leaf tissue from 5 positions (tip, mid, base, mid-lateral, and lateral)
						Leaves 22nd, 22nd, 24th	t1 = 98 and t2 = 126	
					Pointed cabbage	Leaves 15th, 15th, 17th	t1 = 57 and t2 = 85	
						Leaves 17th, 21st, 22nd	t1 = 71 and t2 = 99	
August	90 (3 cultivars × 3 blocks × 10 plants)	3 plants per timepoint per cultivar (1 per block)	Destructive	Leaf morphology and development	Round and pointed cabbage and collard	Leaves	28, 35, 42, 49, 56, 63, 70, and 77	Total number of leaves and their leaf area
					Round and pointed cabbage	Leaf blade	70	Leaf curvature
						Leafy head	-	First heading leaf

**Table 2:** Duration and leaf numbers of the four developmental stages of round and pointed cabbages.

	Stages	Seedling	Rosette	Folding	Heading
Round (Excalibur hybrid variety)	DAS	~10 to ~38	~38 to ~60	~60 to ~70 (latest 84)	~70 to cracking
	Duration (days)	~28	~22	~10	>56
	Leaf number	1st to 7th	8th to ~15th	~16th to ~23rd (latest 24th)	~24th to …
Pointed (Sonsma hybrid variety)	DAS	~8 to ~33	~33 to ~48	~48 to ~56 (latest 84)	~56 to cracking
	Duration (days)	~25	~15	~8	>56
	Leaf number	1st to 6th	7th to ~14th	~15th to ~17th (latest 21st)	~18th to …

**Table 3:** The difference between heading and rosette leaves in the ratio of palisade-to-spongy parenchyma (P/S) for the cellular parameters at both t1 and t2, and the ratio of the change in palisade to the change in spongy parenchyma from t1 to t2 (∆P/∆S = (P_t2_ − P_t1_)/(S_t2_ − S_t1_)). The original data for each biological replicate are provided in Table S7.

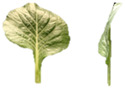 **Rosette** Round		P/S (t1)	P/S (t2)	∆P/∆S
	**Tissue Thickness**	**1.00**	**1.00**	**1.00**
	Single Cell Area	1.02	1.36	1.48
	Cell Density	1.02	1.15	-0.24
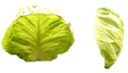 **Heading** Round		P/S (t1)	P/S (t2)	∆P/∆S
	**Tissue Thickness**	**0.88**	**0.86**	**0.79**
	Single Cell Area	1.23	1.21	1.17
	Cell Density	1.05	1.08	0.46
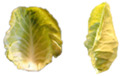 **Heading** Pointed		P/S (t1)	P/S (t2)	∆P/∆S
	**Tissue Thickness**	**0.79**	**0.79**	**0.74**
	Single Cell Area	1.03	1.13	1.41
	Cell Density	1.06	1.10	0.50

## Data Availability

Data are contained within the article or Supplementary Materials.

## Supplementary Materials

The following supporting information can be downloaded at: https://www.mdpi.com/article/10.3390/plants13050656/s1, Table S1: Number of leaves at different days after sowing (DAS) for the different *B. olareacea* cultivars; Table S2: Leaf area across three *B. olareacea* cultivars at different timepoints (days after sowing); Table S3: Curvature ratio of pointed and round cabbage leaves at 70 DAS (days after sowing); Table S4: Leaf shape analysis; Table S5: The first heading leaf of pointed and round cultivars scored at different time points and plants; Table S6: Leaf histological parameters; Table S7: Palisade-to-spongy parenchyma (P/S) ratio of separate leaf positions for different cellular parameters; Table S8: Palisade-to-spongy parenchyma (P/S) ratio of separate leaf positions for different cellular parameters.
